# Psychometric evidence of a science, technology, engineering, and mathematics career interest survey of Indonesian high school students

**DOI:** 10.1038/s41598-025-92587-4

**Published:** 2025-03-07

**Authors:** Ijtihadi Kamilia Amalina, Tibor Vidákovich, Win Phyu Thwe

**Affiliations:** 1 Doctoral School of Education, University of Szeged, Szeged, 6722 Hungary; 2 Center of Educational Technologies, Azerbaijan State University of Economics, 1001 Baku, Azerbaijan; 3 University of Szeged, Institute of Education, Szeged, 6722 Hungary; 4 Education Studies Department, Pyay Education Degree College, Pyay, 08153 Myanmar

**Keywords:** Adaptation, Career interest, Questionnaire, Reliability, STEM, Validity, Psychology, Human behaviour

## Abstract

The disparity between the growth of science, technology, engineering, and mathematics (STEM) job demand and students graduating from STEM areas raises an issue regarding the reason for low interest in STEM careers. An assessment tool is required to investigate this issue. However, the generalizability of existing assessment tools to be conducted cross-culturally becomes a concern. This study aims to report the psychometric evidence of the STEM career interest survey (STEM-CIS) in the Indonesian context using a quantitative design with a stratified random sampling technique. Data from 738 high school students were analyzed using confirmatory factor analysis (CFA). The adapted STEM-CIS showed good psychometric evidence as a single measure, a discipline-specific measure, and a social cognitive career theory (SCCT)-specific subscale measure. The reliability values of the adapted STEM-CIS indicated high, confirming its robustness for assessing STEM career interest among Indonesian high school population. These findings support the use of the adapted STEM-CIS as a contextually relevant and validated tool for cross-cultural research on STEM career interest. This study contributes to the global need for culturally adaptable assessment tools.

## Introduction

The challenges of the 21st century boost the development of information and communication technologies that influence the economies of countries^1^. Science technology, engineering, and mathematics (STEM) play a vital role in economic development, human resources, and competitiveness^2,3^. However, there are qualifications needed to work in STEM areas, called 21st-century skills^4^. Therefore, it is necessary to improve the educational system through STEM education to develop expertise in STEM^4,5^.

The need for STEM professionals increases in line with economic growth, yet the number of STEM graduates is declining^5^. One key factor contributing to this decline is low student interest in STEM careers, further exacerbated by achievement gaps, gender disparities, and STEM stereotypes^3,4,6,7^. For example, in 2021, 65% of people aged 18–74 working in STEM occupation in the United States were male, while only 35% were female^8^. A similar phenomenon is observed in Indonesia, where less than 30% of engineering university students are female, and many do not wish to pursue careers in STEM fields, and gender disparities continue in postgraduate STEM education, with males dominating master’s and doctoral programs^9,10^.

This imbalance limits the potential workforce, innovation, and economic development^5^. Since interest plays a critical role in career choices, understanding how and why students become interested in STEM careers and improving students’ knowledge of STEM through various programs are the first steps to address the shortage in the STEM workforce^2,5,11^. Moreover, STEM career interest is a key focus of ongoing education reforms, such as Indonesia’s “Merdeka” curriculum, which integrates STEM education^2,12^. However, to understand students’ STEM career interests and to evaluate STEM programs, effective instruments are crucial^2,5^.

According to our systematic review of articles between 2003 and 2023, there are 48 articles on developing STEM career interest questionnaires that mostly apply the social cognitive career theory (SCCT). For example, Kier et al.^12^ developed a STEM career interest survey (STEM-CIS) in English to measure STEM as a discipline-specific subject for grades 5 to 8 in rural Southeastern United States, and Tyler-Wood^13^ constructed a STEM semantic survey and a science career interest questionnaire in English for grades 6 to 8 in the United States. The SCCT highlights how career interest evolves through the integration of individual, behavioral, and environmental factors, derived from social cognitive theory, which emphasizes the significance of self-referent thinking in guiding human behavior and motivation^14,15^. Key concepts of SCCT are: (1) self-efficacy and outcome expectation directly influence interest and lead to choice goals; (2) self-efficacy indirectly impact interest through outcome expectation; and (3) person inputs and background contextual affordances impact learning experience, leading to self-efficacy and outcome expectation that influence interest^14^. Although STEM-related assessment instruments have been well established, the cultural relevance and validity of these assessments are becoming an emerging concern^5^.

STEM-CIS has been applied to cross-cultural students^2,5,6^. It has been translated into Turkish^6^, Chinese^5^, Indonesian^2^, Kazakhstani and Russian^4^, Dutch^16^ and Malay^17^. Some scholars selected STEM-CIS to be adapted because it measures all STEM disciplines, has a strong theoretical foundation, has well-implemented development stages, has an appropriate sample size in the validation process, and has strong psychometric evidence^2,4–6^. Moreover, it can be applied as a single measure or as a separate discipline-specific measure based on the results of its psychometric analysis^12^.

The issue of generalizability of the STEM-CIS across ages has been considered and has been suggested to be investigated for further study^12^. Most scholars applied STEM-CIS for middle school students^2,6^. Only a few studies adapted STEM-CIS for different age respondents, e.g., Mau et al.^5^ translated it into Chinese and validated it on high school students, and Grimmon et al.^16^ translated it into Dutch for primary school students.

Interest in STEM careers has become a concern in the Asian educational system, including Indonesia, due to the increasing professional demand in STEM fields but the low number of STEM graduates, which is attributed to limited interest^2,4,5,17^. In Indonesia, technology-related occupations were projected to grow from 445,068 to 602,022 jobs between 2016 and 2020, yet the number of STEM graduates remains low at only 8 graduates per 10,000 people^18,19^. Although several studies have reported that Indonesian high school students initially show a high interest in STEM carer, this interest declines over time, as evidenced by the low number of STEM graduates^20,21^. Despite these phenomena, Indonesian government has introduced policies related to STEM education. There is no independent STEM curriculum in Indonesia, but STEM is integrated into the mathematics and science curriculum through problem-solving activities^22,23^. Additionally, STEM is emphasized in several school programs through project-based activities conducted every semester to increase students’ interest in STEM careers^23^.

Given the high demand for STEM professionals and the Indonesian government’s efforts to integrate STEM into the curriculum, alongside the persistently low interest in STEM careers, conducting research on STEM career interest in Indonesia is crucial. However, previous studies have highlighted limited research focused on developing assessment of STEM career interest in the Indonesian context. Ardianto et al.^2^ conducted a study aimed at validating STEM-CIS for Indonesian middle school students. However, there is an issue regarding the method used and whether the translation can be used for high school students. Therefore, our objective is to adapt and report the psychometric evidence of STEM-CIS in Indonesian high school students. The construct validity and reliability will be reported in detail in every discipline-specific subscale and SCCT-subscale.

This study focused on Java and Sumatera, as both have implemented the new “Merdeka” curriculum, which emphasizes STEM integration. However, these regions exhibit significant disparities in regional development, curriculum implementation, and teacher preparedness. Java, as the most developed and populous region, is likely more prepared for the curriculum, while Sumatera, with its geographical challenges, may encounter greater difficulties in implementation. By examining these contrasting areas, this study provides comprehensive evidence that STEM-CIS’s applicability across a diverse range of Indonesian high school students.

## Theoretical background

STEM-CIS was developed according to the SCCT^14^. SCCT is based on Bandura’s social cognitive theory, which argues that the most influential component in setting goals and actions is self-efficacy^15^. SCCT is a model to predict interest and career choice by integrating individual, behavioral, and environmental aspects. The individual aspect of the SCCT focuses on self-efficacy, outcome expectations, interest, and goals. Self-efficacy is beliefs in one’s ability to complete the task or perform a specific behavior. The outcome expectation is beliefs about the consequences of taking particular actions. Self-efficacy and outcome expectation tend to promote interest, and these three variables jointly promote the choice goal. In addition, outcome expectation is also influenced by self-efficacy. In SCCT, the environmental aspect is also considered an influential factor in career interest. Personal characteristics, contextual background, and learning experience impact interest through self-efficacy and outcome expectation.

STEM-CIS was developed in the project that emphasizes careers in the context of classroom instruction in rural areas^12^. It measures self-efficacy, personal goal, outcome expectations, interest, personal input, and contextual support in each STEM discipline. The survey consists of 44 items in total, with 11 items for each discipline, including two items each for self-efficacy, personal goal, outcome expectations, interest, and contextual support subscales, and one item in personal input subscale. Therefore, across the entire questionnaire, there are 8 items each for self-efficacy, personal goal, outcome expectations, interest, and contextual support subscales, and four items for personal input subscale. STEM-CIS was validated in 1,061 students in grades 5–8 in rural southeastern United States of America using confirmatory factor analysis (CFA). It was validated in a four-factor full scale and in a separate discipline-specific subscale. The results showed that all models had good fit indexes and acceptable factor loading values. Therefore, STEM-CIS can be used as a single measure and as discipline-specific measures.

Several studies adapted STEM-CIS cross-culturally and reported psychometric evidence, e.g., Turkish version^6^, Chinese version^5^, Indonesian version^2^, and Dutch version^16^. However, only Mau et al.^5^ and Grimmon et al.^16^ administered the adapted questionnaire to different targeted respondent groups as in the original study, which were in high school and primary school, respectively.

Ünlü et al.^6^ and Mau et al.^5^ adapted the STEM-CIS into their respective languages using CFA, Turkish and Chinese, respectively. However, they targeted different respondent groups. Ünlü et al.^6^ validated the instrument with a sample of 1,033 Turkish middle school students in grades 5 to 8, while Mau et al.^5^ validated it with 590 high school students in Taiwan. Ünlü et al.^6^ removed item 11 from each discipline in the original questionnaire (e.g., I know someone in my family who applies mathematics in their job) during the cultural and language adaptation process because these items were not appropriate for Turkish culture, could cause contradictions in terms, and did not serve the purpose of the assessment. In contrast, Mau et al.^5^ excluded item 11 in all disciplines due to low factor loading values. Both studies finalized the questionnaire with 40 items based on CFA, which showed good factor loading values.

The models of discipline-specific subscales in Ünlü et al.^6^ had good fit indexes, with some low fit indexes in science (normed fit index (NFI) = 0.83 and comparative fit index (CFI) = 0.83), technology (NFI = 0.86), and mathematics (NFI = 0.85 and CFI = 0.88). Reliability values on all subscales were acceptable. However, the worst factor loading value and reliability were on the science subscale. Unlike Ünlü et al.^6^, Mau et al.^5^ provided a more detailed psychometric evidence of the adapted STEM-CIS. All models met the cutoff criteria for the fit indexes (CFI and Tucker–Lewis index (TLI) greater than 0.90 and root mean squared error of approximation (RMSEA) and standardized root mean square residual (SRMR) less than 0.08), including four- factor full scale, six-factor full scale, discipline-specific subscale, and SCCT subscale. The reliability values in every subscale were more than 0.90, and the reliability value in the overall questionnaire was 0.94.

Ardianto et al.^2^ conducted a study on 572 Indonesian middle school students. According to the translation process, four items were excluded due to cultural and language problems based on experts’ evaluations; however, there is no specific information regarding the deleted items. Unlike other studies that employed CFA, this study used Rasch analysis and revealed that three items did not meet the item fit criteria. In addition, there were grade and gender biases in some items. The Cronbach’s alpha value obtained from the test result was 0.92. However, Rasch analysis is considered inappropriate to be used to validate the construct of the questionnaire.

The 44-item STEM-CIS was also adapted for Dutch aged ten to twelve, a different respondent group from the original STEM-CIS and other relevant studies^2,5,6,16^. The instrument was first tested in two pilot studies, and after revision, the instrument was administered to 212 students. Cronbach’s alpha value indicated good with 0.91. However, there was no information on the construct validity of the instrument.

Some scholars applied STEM-CIS in only a specific discipline or subscale, e.g., self-efficacy and interest subscales^24^, and contextual support and interest subscales^25^. Other scholars translated and applied STEM-CIS but did not report detailed psychometric evidence, e.g., the Kazakhstani version^4,26^, the Russian version^4^, and the Malay version^17^. In addition, some studies employed the Turkish version of the STEM-CIS by Ünlü et al.^6^ with acceptable reliabilities and fit models^1,11,27,28^.

All studies adapting STEM-CIS conducted a cultural and language adaptation process prior to validating the construct to ensure that the content and language of the instrument were relevant to the respondents^2,5,6^. Ardianto et al.^2^ adapted the STEM-CIS to the Indonesian context and language, and it was evaluated by three experts from science and Indonesian language. Unlu et al.^6^ adapted the STEM-CIS to the Turkish context and language, and it was examined by 12 experts in language and education. The revised version was then evaluated and translated back into English by three Turkish experts to ensure that the meaning remain unchanged. Mau et al.^5^ translated the STEM-CIS into Chinese, which was translated back by two authors in education. The instrument was then administered to five high school students to ensure the readability and understanding. This process is crucial to avoid misinterpretation of items in different contexts, biased response, reduced validity and reliability, ethical issues, and poor decision-making.

## Methods

### Respondents

This study employed a stratified random sampling method by established criteria for selecting schools and randomly chose both schools, that satisfied the criteria, and students based on their and their parents’ consents. The study was carried out in Indonesian high schools in the provinces of Java and Sumatera, from both urban and rural areas. These provinces were selected because they have implemented the new curriculum called “Merdeka” curriculum, which emphasizes and integrates STEM education. Schools were selected based on their application of the “Merdeka” curriculum and their A-accreditation status. A-accreditation is a crucial criterion as it represents the schools’ commitment to the “Merdeka” curriculum. There are 3,912 schools in Java and Sumatera that have A-accreditation and implement the “Merdeka” curriculum, with the total number of 1,173,600 students in grade 10 and 11. These schools include public, private, and vocational schools. Given the total population, with a confidence level of 99% and margin of error of 5%, the minimum sample required for the study is 666. The study included 738 students, making the sample representative of the population.

High school in Indonesia includes grades 10 to 12 and consists of private, public, and vocational schools. Students are required to select a major at the beginning of high school based on their entrance test scores and interests. In this study, “major” refers to the field of specialization chosen by students in high school. Public and private schools offer limited major options: social and language (non-STEM), and science (STEM). Vocational schools offer a wide variety of majors, including non-STEM field (e.g., music, hospitality) and STEM field (mechanical engineering, software engineering, pharmacy). Students who select a STEM major primarily study STEM-related subjects, such as physics, chemistry and advanced mathematics. This study included respondents from grades 10 and 11, as grade 12 students are already focused on selecting their university major fields, making it less appropriate to include them in the study, as their response may reflect their current preparation for specific university program rather than genuine interest in STEM careers. Additionally, grade 12 students are typically preoccupied with preparing for final exam and university entrance tests, leaving them with less time and energy to participate in research study. Table 1 provides the detailed characteristic of the respondents.

**Table 1:** Demographic characteristics of the respondents.

Demographic characteristics		*N*	%
Grades	10 (M age = 15.53, SD = 0.59)	288	39.02
	11 (M age = 16.52, SD = 0.57)	450	60.98
Gender	Male	289	38.80
	Female	452	61.20
School type	Public school	544	73.70
	Private school	152	20.60
	Vocational school	42	5.70
Major	STEM	502	68.02
	Non-STEM	236	31.98
School location	Urban	313	42.41
	Rural	425	57.59
Ethnicity	Javanese	641	86.90
	Malay	66	8.90
	Others	31	4.20

### Instrument

The instrument applied in the study is the adapted STEM-CIS^12^. The instrument uses a 5-point Likert scale that has a total of 44 items, with 11 items in every discipline-specific subscale. It includes the subscales of science, mathematics, engineering, and technology. Each of the subscales measures self-efficacy (*n* = 2), personal goal (*n* = 2), outcome expectation (*n* = 2), interest in the discipline (*n* = 2), contextual support (*n* = 2), and personal input (*n* = 1). Hence the total number of items measuring each construct—self-efficacy, outcome expectation, personal goal, interest and contextual support— is 8, while the total number of items measuring personal input is 4. The questionnaire comprises instructions, statements, and demographic information.

The meanings of terms related to science, technology, engineering, and mathematics, with career associated with these disciplines, are explained in the instruction section of the questionnaire. Mathematics involves the study of numbers, quantities, shapes, patterns, and their relationships, while science focuses on understanding the natural and physical world through observation, experimentation, and analysis. Careers related to science and mathematics include roles that apply these concepts, such as astronomer, chemist, data scientist, statistician, etc. Technology pertains to the creation of tools, machines, or systems designed to simplify and improve life, with career examples including software developers and IT support specialist. Engineering involves designing and constructing things by implementing science and mathematics principles, with careers such as civil engineer and mechanical engineer serving as examples. Table 2 provides examples of items in each SCCT category within science subscale. The items in the mathematics, technology, and engineering subscales are similar to those in science subscale.

**Table 2:** Example of items in STEM-CIS in science subscale.

SCCT category	Example of items
Self-efficacy	I am able to get a good grade in my science class
Personal goal	I plan to use science in my future career
Outcome expectation	If I do well in science classes, it will help me in my future career
Interest	I am interested in careers that use science
Contextual support	I have a role model in a science career
Personal input	I would feel comfortable talking to people who work in science careers

### Procedure

The adaptation process began by reviewing existing questionnaires related to STEM career interests and selecting the most suitable one for adaptation. A team of ten experts, including educators, university professors, and researchers, all with over seven years of experience and at least a master’s degree, contributed to the adaptation process. Their expertise spans education, STEM fields, and language, ensuring a well-rounded approach to the adaptation.

The selected questionnaire was adapted to the Indonesian context by two Indonesian experts in education: a corresponding author, who is a researcher in STEM education, and a mathematics education lecturer. The adapted questionnaire was then independently translated into Indonesian by four Indonesian native speakers: a corresponding author (researcher in STEM education) and three STEM educators, considering the Indonesian cultures, conditions, curriculum and the background of the students. The translated questionnaire was reviewed by two university professors in the field of education and one lecturer in language studies. Subsequently, the questionnaire was translated back into English by two experts: (1) an Indonesian female Ph.D. student in English education; and (2) an Indonesian male lecturer in mathematics education. The corresponding author, a native Indonesian STEM researcher, revised the translation to address any statements that altered the original meaning. The back translation process ensures that the adaptation and translation do not change the meaning of the original questionnaire. Finally, the questionnaire was administered to five Indonesian high school students to assess its readability and understanding. The language used to collect information was Indonesian.

The STEM-CIS is adapted and translated according to the characteristics of the respondents and the conditions in Indonesia (e.g., curriculum, etc.). In high school, science is divided into physics, chemistry, and biology. Hence, first, it was defined science as a subject that includes all three mentioned subjects. Some words were difficult to be translated into the Indonesian language because of language limitation. For example, “engineer” is translated into “the person who works in an engineering sector”. Another issue was regarding plural and singular words. In the Indonesian language, it is needed to repeat the word to represent the plural word.

After being translated into Indonesian and reviewed by experts, two experts translated it back into English. After doing the back-translation, there were two items that need to be revised. These translated items had different meaning from the original items. The item “I am able to do well in activities that involve technology” was translated into “I can work using technology well”. Moreover, the item “I am able to do well in activities that involve engineering” was translated into “I can do work that requires engineering”. Therefore, these items were needed to be revised to fit the original version by discussing with the expert translators. After revising these items, the adapted STEM-CIS can be administered to the five students to assess its readability and understanding, then administered to target sample to analyze the psychometric evidence. After cultural and language adaptation process, the corresponding author submitted ethical approval from the Institutional Review Board of the University of Szeged under reference number 7/2022 and coordinated with several schools. Participating schools were required to confirm their willingness to participate within two weeks after receiving ethical approval. The participating schools sent written consent to parents or guardians of students that provided detailed information about the study, including its purpose, procedure, and data confidentiality. After receiving written consent, the schools generated a list of eligible respondents and shared it with the corresponding author. The corresponding author discussed with mathematics teachers to deliver the questionnaire. Before completing the questionnaire, the corresponding author and teachers requested verbal consent from the students emphasizing the voluntary nature of participation. Students were informed that they could withdraw from the study at any time with no consequences. A 25-minute voluntary computer-based questionnaire was delivered during the teaching and learning process. Data collection was carried out between November 22, 2023 and January 30, 2024. All research was performed in accordance with relevant guidelines/regulations. The data obtained were analyzed to report the psychometric evidence of the adapted questionnaire.

### Data analysis

Data were analyzed to report the construct validity and reliability. CFA was used by applying the Mplus8 version 8.4 application with the WLSMV estimator and theta parameterization to test the construct validity. CFA is an appropriate method for ensuring that the construct of the original questionnaire remains valid, as applied by previous relevant studies^5,6^. To assess model fit, Chi-square values, CFI, RMSEA, TLI, SRMR, and the factor loadings in each item were examined. These fit indexes were selected because they have been commonly used in previous studies validating the STEM-CIS^5,6,12^. Specifically, CFI and TLI assess comparative fit, while RMSEA and SRMR evaluate absolute fit, providing a comprehensive measure of model performance while accounting for sample size and model complexity^5,29^.

CFA was performed on discipline-specific subscales (e.g., mathematics, science, engineering, and technology), four-factor full scale, and SCCT subscales (e.g. self-efficacy, personal goal, outcome expectation, etc.). The CFI and TLI values were classified as good if they were greater than or equal to 0.95; however, they were still acceptable with a value of approximately 0.90^29^. Additionally, a good SRMR value was less than 0.08^29^, and the RMSEA value was considered acceptable if it was less than 0.08, marginal if it was in the range [0.08, 0.10], and mediocre if it was more than 0.10^30^. A model is considered a good fit if it meets the cutoff criteria for fit indexes: CFI and TLI greater than 0.90, and SRMR and RMSEA less than 0.08. The cut-off point for factor loading is 0.4 based on the sample size of this study^29^. To find the best model, model comparison and index modification were performed. Index modification was executed by correlating potential items based on the value of the modification indexes. Moreover, Cronbach’s alpha was utilized to test reliability using IBM SPSS Statistics 25.

## Results

### The construct validity of the adapted STEM-CIS

Kaiser-Meyer-Olkin (KMO) analysis was performed prior to CFA analysis. The result of KMO was 0.949, indicating that the sample was adequate to conduct CFA and the data were well-suited for factor analysis. The results of CFA with four subscales from categorical data informed the fit of the model, as the CFI and TLI greater than 0.90, and SRMR and RMSEA less than 0.08 (see Model 2 in Table 3). However, the model comparison must be performed to ensure the effect of the dimensions or subscales. Therefore, a model comparison was conducted between Model 1 (one dimension) and Model 2 (four dimensions). Each model consists of 44 items, but the items in Model 2 are categorized into four dimensions (i.e., science, technology, engineering, and mathematics), with 11 items in each dimension. The results revealed that the Chi-square test for different testing was significant χ^2^ (12) = 2151.786, *p* < .001. It showed that there is a significant difference between Model 1 and Model 2. The best model was Model 2 since it had better fit indexes. Table 3 explains the model comparison of the adapted STEM-CIS.

**Table 3:** Model comparison of the adapted STEM-CIS.

Fit indexes	Model 1	Model 2
Parameter estimated	220	323
Chi square	$\:{\chi\:}^{2}$ (902) = 17096.580, *p* < .001	$\:{\chi\:}^{2}$ (890) = 3741.123, *p* < .001
CFI	0.654	0.939
TLI	0.638	0.935
RMSEA	0.156	0.066
SRMR	0.157	0.053

Model 2 with four subscales had acceptable fit indexes, indicating that it fits the observed data well. Hence, the model can be used to perform the factor loading. The statistical test to obtain factor loadings was confirmatory factor analysis. All items had acceptable factor loading values between 0.60 and 0.92. This shows that all items had sufficiently strong correlation with STEM career interest construct and contributed meaningfully to its measurement. The items that had the lowest and highest factor loading values were item 11 in the mathematics subscale (factor loading = 0.606) and item 4 in the technology subscale (factor loading = 0.918). The findings indicate that students strongly associate item 4 in the technology subscale with their interest in technology-related careers. Table 4 performs the factor loading values of the items on each subscale when all four factors were analyzed together.

**Table 4:** Factor loading values of adapted STEM-CIS in four-factor full scale.

Item	Science		Mathematics		Technology		Engineering	
	Factor loading	*R*-square	Factor loading	*R*-square	Factor loading	*R*-square	Factor loading	*R*-square
1	0.695	0.483	0.684	0.468	0.769	0.591	0.815	0.664
2	0.717	0.513	0.719	0.517	0.754	0.569	0.971	0.759
3	0.818	0.669	0.829	0.687	0.869	0.755	0.924	0.854
4	0.817	0.668	0.815	0.664	0.918	0.843	0.896	0.803
5	0.789	0.623	0.785	0.616	0.862	0.744	0.845	0.714
6	0.693	0.481	0.736	0.541	0.735	0.540	0.820	0.673
7	0.823	0.677	0.832	0.692	0.700	0.489	0.916	0.840
8	0.802	0.644	0.773	0.597	0.838	0.703	0.903	0.816
9	0.774	0.598	0.736	0.542	0.793	0.628	0.809	0.654
10	0.787	0.619	0.778	0.605	0.796	0.633	0.769	0.592
11	0.616	0.380	0.606	0.368	0.673	0.453	0.616	0.379

#### Discipline-specific subscale

##### Science subscale

The results of the model fit indexes on the science subscale were acceptable with χ2(44) = 642.015, *p* < .001, RMSEA = 0.136, CFI = 0.951, TLI = 0.939, and SRMR = 0.040. These results indicate that the model for the science subscale fits the observed data well within the Indonesian high school context. However, the RMSEA result was relatively large. Therefore, it is needed to increase the fit indexes by correlating the potential items. It can be considered from the modification indexes. The decision to correlate potential items was based on their content similarities, as well as their conceptual connections within the context of STEM career interest. These correlations potentially improve model fit and were made to refine the measurement model without changing the theoretical framework of the study.

The results revealed that correlating item 1 with item 2 resulted in a 175.787 increase in the modification index (*r* = .3886), correlating item 9 with items 10 and 11 resulted in an increase of 85.925 (*r* = .309) and 85.060 (*r* = .301) in the modification indexes, respectively, and correlating item 3 with item 7 led to a 43.899 increase in the modification index (*r* = .240). As a result of correlating some potential items, the fit indexes of the science subscale increased and were acceptable, specifically the RMSEA value. These findings suggest that item 1 shared content similarity with item 2, item 9 with items 10 and 11, and item 3 with item 7. Most of these correlated items belong to the same subscale, such as items 1 and 2 are part of self-efficacy subscale, while items 9 and 11 fall under the contextual support subscale. Therefore, it is reasonable that the CFA results indicated these items should be correlated due to their conceptual similarity. Table 5 describes the values of the fit indexes in some models. The factor loading values for all items on the science subscale were acceptable, indicating all items contributed meaningfully to measure science career interest, with the lowest of 0.547 (item 11) and the highest of 0.845 (item 4).

The study employed an analysis in every discipline-specific subscale based on SCCT subscales. However, the personal input subscale could not be included in the analysis since it consists only an item in each of the discipline. Hence, item 10 was excluded from all disciplines that measure personal input in our analysis (e.g., I would feel comfortable talking to people who work in science careers). The results for science discipline revealed the model fit in all fit indexes category, suggesting that the science career interest model based on SCCT subscales aligned well with the observed data (χ2(24) = 113.385, *p* < .001, RMSEA = 0.079, CFI = 0.990, TLI = 0.981 and SRMR = 0.019). The factor loading values were high between 0.732 and 0.864, confirming that all items contributed meaningfully to measure science career interest based on SCCT subscales.

##### Mathematics subscale

The mathematics subscale had an inappropriate RMSEA value (χ2(55) = 940.409, *p* < .001, RMSEA = 0.166, CFI = 0.928, TLI = 0.910 and SRMR = 0.049). Item 2 was correlated with items 1 and 11 (increase modification index by 322.984, *r* = .469, and increase modification index by 41.225, *r* = -.175), item 9 with items 10 and 11 (increase modification index by 91.245, *r* = .329, and increase modification index by 110.221, *r* = .309), and item 5 with items 3 and 4 (increase modification index by 54.365, *r* = .328, and increase modification index by 51.703, *r* = .313). These correlations were justified by their shared constructs, such as items 1 and 2 belonging to the self-efficacy subscale and items 3 and 4 to the personal goal subscale. As a result, there was an increase in the fit indexes values (see Table 5). However, the RMSEA value was still large, indicating some localized misfit. Although, other fit indexes supported model adequacy and the factor loading values of all items indicated good, ranged from 0.536 to 0.829. Therefore, the final model for the mathematics subscale remains acceptable and all the items contribute meaningfully to assess mathematics career interest. The model of mathematics discipline based on SCCT subscales revealed fit (χ2(24) = 133.866, *p* < .001, RMSEA = 0.079, CFI = 0.990, TLI = 0.981 and SRMR = 0.017). The factor loading values ranged from 0.632 (item 11) to 0.89 (item 9). These results indicate that the mathematics career interest model based on SCCT subscales fits the observed data well, with all items playing a significant role in assessing mathematics career interest based on SCCT subscales.

##### Technology subscale

The technology subscale had a problem with the value of the RMSEA, but other fit index values were acceptable (χ2(44) = 887.538, *p* < .001, RMSEA = 0.161, CFI = 0.939, TLI = 0.924 and SRMR = 0.045). To increase the values of the fit indexes, item 1 was correlated with item 2 which led to an increase of 274.308 in the modification index (*r* = .016). Furthermore, correlating item 4 with item 5 and item 6 with item 7 resulted in an increase of 67.832 (*r* = .285) and 98.192 (*r* = .273) in the modification indexes, respectively. Other correlations were between item 9 and items 8 and 11, which increased the modification indexes of 92.870 (*r* = .284) and 92.474 (*r* = .359), respectively. These items were suggested to be correlated because they belong to similar constructs, such as items 5 and 6 belong to the outcome expectation subscale. After correlating these items, the fit index values of the technology subscale inclined, but did not solve the problem of the RMSEA value (see Table 5). However, the factor loading values for all items were acceptable [0.515; 0.913]. These results indicate that the technology career interest subscale still effectively supports the observed data, with all items contributing significantly to the measurement of technology career interest. The result of the fit indexes in the technology discipline based on SCCT subscales revealed the fit model (χ2(24) = 138.677, *p* < .001, RMSEA = 0.081, CFI = 0.993, TLI = 0.987 and SRMR = 0.015). The highest factor loading value was 0.927 (item 4) and the lowest loading factor value was 0.695 (item 11). These findings suggest that the technology career interest based on SCCT subscales model aligns well with the observed data, with all items contributing effectively to measure technology career interest based on SCCT subscales.

##### Engineering subscale

**Fig. 1:**
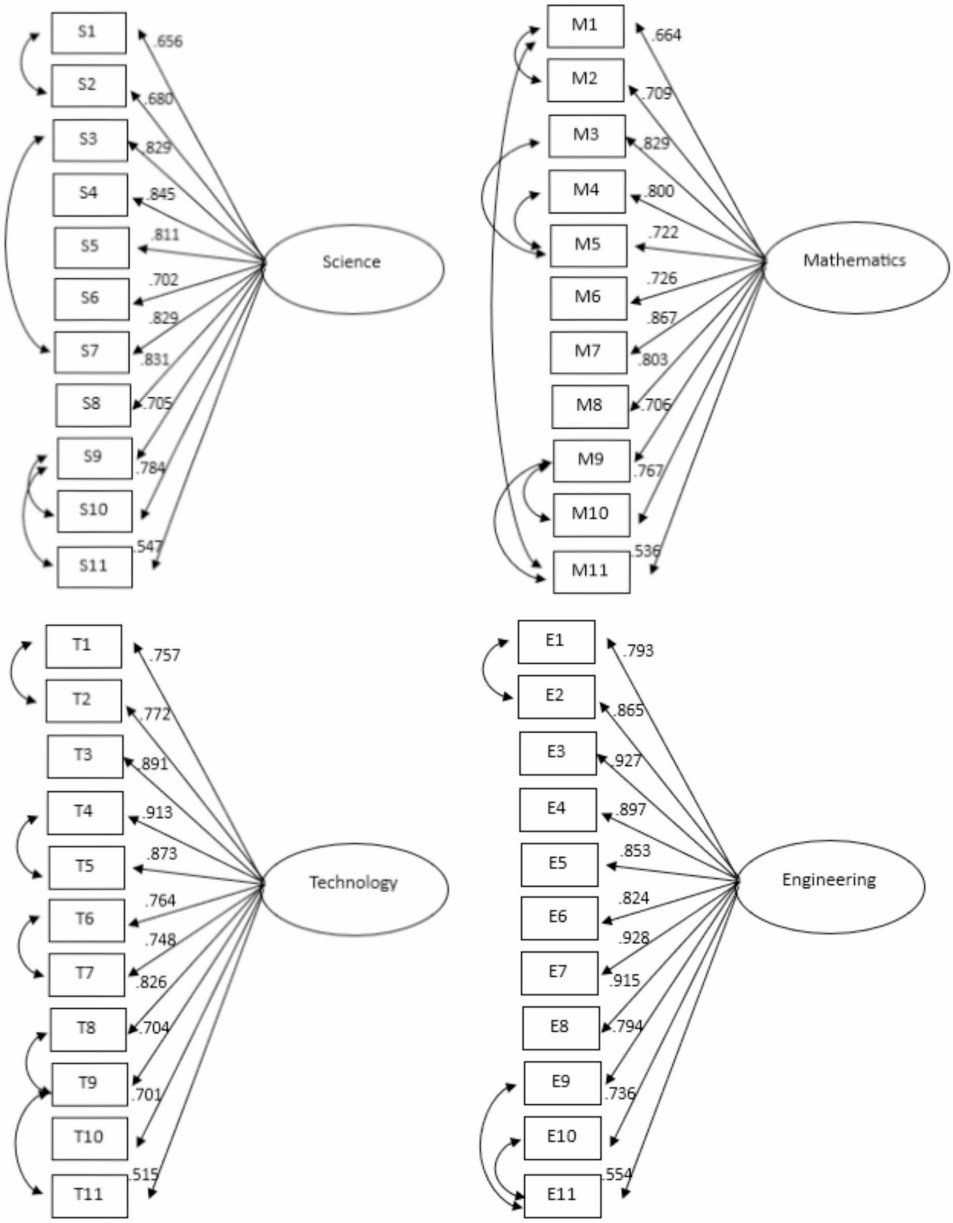
The model of the adapted STEM-CIS based on discipline-specific subscales.

Similarly to the mathematics and technology subscales, the engineering subscale had the issue related to the RMSEA value (χ2(44) = 1278.227, *p* < .001, RMSEA = 0.195, CFI = 0.966, TLI = 0.958, and SRMR = 0.038). The correlation between item 1 and item 2 increased the modification index of 529.985 (*r* = .568). Furthermore, the correlation of item 11 with items 9 and 10 resulted in an increase of 146.043 (*r* = .383) and 258.800 (*r* = .419) in the modification indexes, respectively. These items were suggested to be correlated due to their similar constructs, such as items 1 and 2 belong to the self-efficacy subscale. It produced better fit index values, but there was still a problem with the RMSEA value (see Table 5). The factor loading values for all items range from 0.554 to 0.913, indicating acceptable values. Figure 1 illustrates the STEM-CIS model based on discipline-specific subscales. Similarly to other disciplines, in the engineering model using SCCT subscales, the results showed a model fit (χ2(24) = 106.448, *p* < .001, RMSEA = 0.068, CFI = 0.998, TLI = 0.996 and SRMR = 0.008). The loading factor values were in the range of 0.669 (item 2) and 0.970 (item 11). These findings indicate that the engineering career interest models, both overall and based on SCCT subscales, align well with the observed data, with all items contributing effectively to assess engineering career interest in general and according to the SCCT subscales.

#### SCCT subscales

**Fig. 2:**
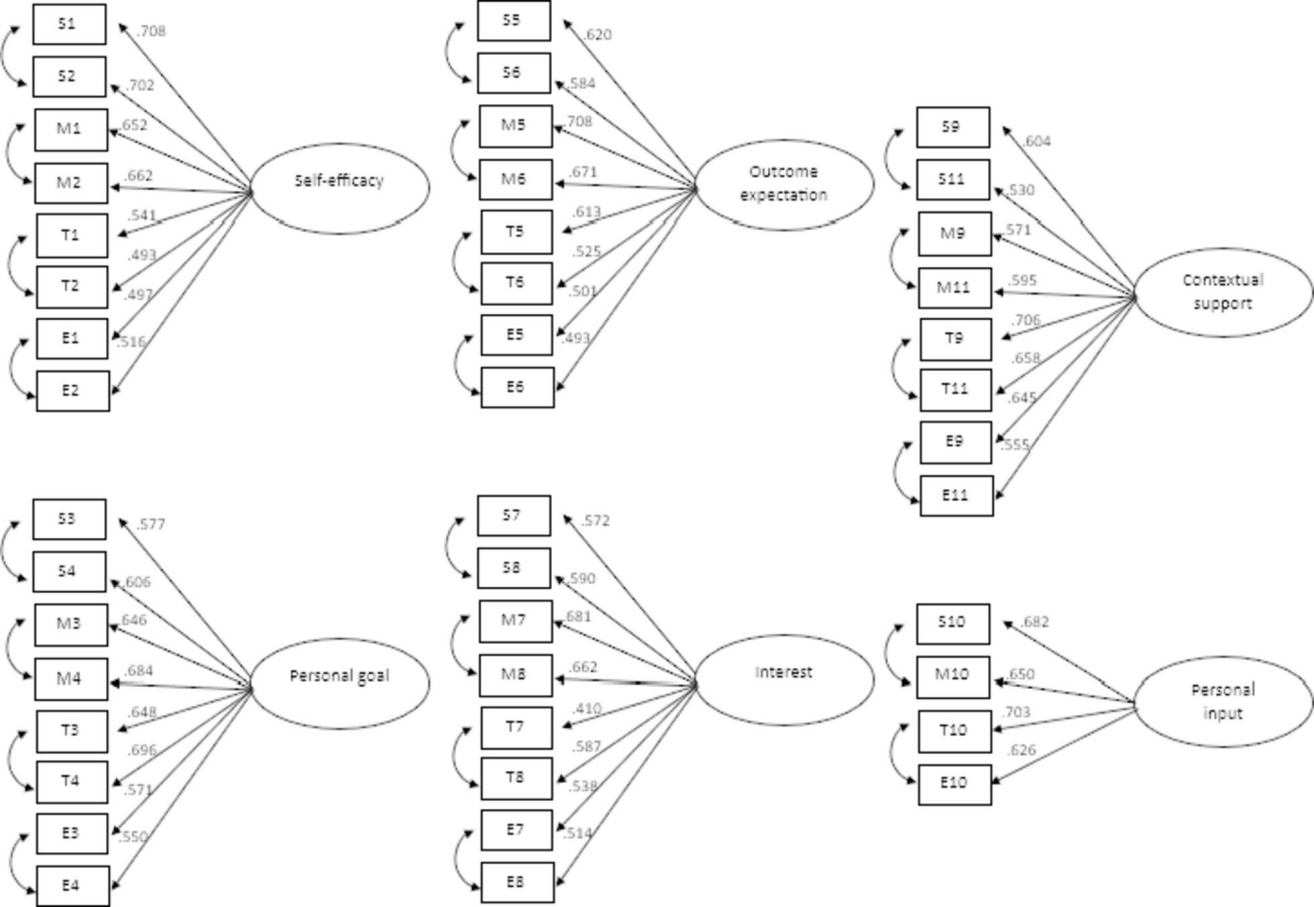
The model of the adapted STEM-CIS based on the SCCT subscale.

Models of self-efficacy, personal goal, outcome expectation, and interest subscales had problems with the RMSEA values. However, all fit index categories were acceptable. The modification indexes have been performed but did not solve the problem. Furthermore, the model of contextual support and personal input subscales had issues with respect to the TLI and RMSEA values (see Table 5 for detailed results). Despite the problems with RMSEA or TLI, the factor loadings on all subscales were higher than 0.40. The lowest factor loading value was on the interest subscale in the technology discipline (0.410), and the highest factor loading values were on self-efficacy in the science discipline and the outcome expectation in the mathematics discipline (0.708). These findings indicate that all items contributed effectively to evaluate self-efficacy, personal goals, outcome expectations, interest, contextual support, and personal input. Figure 2 illustrates the factor loading values on the SCCT subscales.

**Table 5:** The fit indexes values of the STEM-CIS model.

	Parameter estimated	Chi square	CFI	TLI	RMSEA	SRMR
Single-factor full scale	220	$\:{\chi\:}^{2}$ (902) = 17096.58, *p* < .001	0.654	0.638	0.156	0.157
Discipline-specific subscales						
Four-factor full scale	232	$\:{\chi\:}^{2}$ (890) = 3741.123, *p* < .001	0.939	0.935	0.066	0.053
Science only	59	$\:{\chi\:}^{2}$ (40) = 279.939, *p* < .001	0.980	0.973	0.090	0.027
Mathematics only	61	$\:{\chi\:}^{2}$ (38) = 350.361, *p* < .001	0.975	0.964	0.106	0.030
Technology only	56	$\:{\chi\:}^{2}$ (39) = 376.373, *p* < .001	0.976	0.966	0.108	0.032
Engineering only	58	$\:{\chi\:}^{2}$ (41) = 399.016, *p* < .001	0.990	0.987	0.109	0.018
SCCT-subscales						
Self-efficacy	44	$\:{\chi\:}^{2}$ (16) = 480.417, *p* < .001	0.952	0.916	0.198	0.071
Personal goal	44	$\:{\chi\:}^{2}$ (16) = 225.774, *p* < .001	0.975	0.957	0.133	0.048
Outcome expectation	44	$\:{\chi\:}^{2}$ (16) = 222.255, *p* < .001	0.951	0.914	0.132	0.047
Interest	44	$\:{\chi\:}^{2}$ (16) = 264.957, *p* < .001	0.975	0.957	0.145	0.052
Contextual support	44	$\:{\chi\:}^{2}$ (16) = 291.944, *p* < .001	0.929	0.876	0.153	0.043
Personal input	20	$\:{\chi\:}^{2}$ (2) = 117.091, *p* < .001	0.908	0.723	0.279	0.044
Discipline-specific based on SCCT-subscales (without personal input subscale)						
Science	61	$\:{\chi\:}^{2}$ (24) = 133.385, *p* < .001	0.990	0.981	0.079	0.019
Mathematics	61	$\:{\chi\:}^{2}$ (24) = 133.866, *p* < .001	0.990	0.981	0.079	0.017
Technology	61	$\:{\chi\:}^{2}$ (24) = 138.677, *p* < .001	0.993	0.987	0.081	0.015
Engineering	61	$\:{\chi\:}^{2}$ (24) = 106.448, *p* < .001	0.998	0.996	0.068	0.008

### The reliability of the adapted STEM-CIS

The reliability of the adapted STEM-CIS indicated a very good reliability (α = 0.950), suggesting that the responses to the STEM-CIS items are consistent and trustworthy for measuring STEM career interest. According to the discipline-specific subscale, it showed that their reliability values were acceptable with 0.898, 0.901, 0.918, and 0.946 for science, mathematics, technology, and engineering, respectively. This shows that the items in each discipline-specific subscale consistently measure the same construct (e.g., the items in the technology career interest subscale measure the same construct of interest in a technology career), but the science subscale had the lowest reliability value compared to the others. It was also calculated the reliability based on SCCT subscales. It revealed slightly low reliability values, but they were still acceptable. The reliability values of self-efficacy, personal goal, outcome expectation, interest, contextual support, and personal input subscales were 0.763, 0.804, 0.774, 0.791, 0.796, and 0.679, respectively. The personal input subscale had the lowest reliability value, indicating that the items in this subscale were slightly less consistent in measuring the intended construct compared to the other subscales.

## Discussion

The STEM-CIS is proven to be used to collect reliable data from Indonesian population in high school contexts. The results of the fit indexes reported that the four-factor full scale was significantly better than the single-factor full scale, indicating an important role for dimensions. The model of single-factor full scale did not exceed the cut-off point, indicating that the model did not fit the observed data of the Indonesian high school context well^29^. This result contradicts the study of Kier et al.^12^ stated that the model of single-factor full scale was better, but not significant, than the four-factor full scale and the study of Mau et al.^5^ revealed that the results between both models were similar. All the factor loading values on the four-factor full scale in this study were acceptable^29^, in the range of 0.60 and 0.92. These findings show that all items on the four-full scale contribute meaningfully to assess STEM career interest for Indonesian high school students. It is similar to the results of Mau et al.^5^ reported that the factor loading values were in the range of 0.67 and 0.88.

The results of the fit indexes based on the discipline-specific subscale and the SCCT subscale indicated the fit models, showing that the adapted STEM-CIS, both the discipline-specific and SCCT subscales, aligns well with the theoretical structure of STEM career interest in the Indonesian high school context. This indicates that the instrument effectively captures students’ discipline-specific career interest and SCCT subscales. The Chi-square results in this study were significant or poor model. However, in practice, Chi-square is not considered to be a very useful index because: (1) Chi-square is affected by sample size, larger samples produce larger and significant Chi-square, (2) Chi-square is affected by model size, model with more variables tends to have larger and significant Chi-square, and (3) Chi-square is affected by the distribution of variables^31^. In this study, the sample size is large and the models have more variables; hence, the Chi-square results were omitted. Another case is where the RMSEA values were classified as marginal and mediocre^30^. RMSEA deals with model complexity, if the model has few degrees of freedom, it has a large probability to have bad RMSEA. With the small number of degrees of freedom in the model, RMSEA is not meaningful and therefore the decision on acceptance of the model is based on CFI and SRMR^32^. Since the degree of freedoms of the discipline-specific subscale models and SCCT subscale models were small in this study, we neglected the results of RMSEA and concluded that the models were fit based on CFI and SRMR.

The factor loading values in four disciplines were indicated as acceptable, indicating that all the items made a meaningful contribution to measuring career interest in these fields among Indonesian high school students. These factor loadings in this study were slightly worse compared to previous studies with the same age group, which ranged from 0.70 to 0.93^5^. However, the factor loading values in this study were better than the original STEM-CIS, but were similar in the technology subscale^12^, and worse in the engineering subscale in another study^6^. These variations may be influenced by contextual factors such as differences in STEM education curricula, students’ exposure to STEM-related activities, and societal perceptions of STEM careers in Indonesia. The findings highlight the need to consider local educational and cultural contexts when adapting career interest measurements.

The discipline-specific subscale models were also analyzed on the basis of the construct on the SCCT aspects. The results revealed that when all six SCCT subscales in the discipline-specific models were included, there were problems with the analyses. However, when the personal input subscale was excluded from the model, all four disciplines had fit model. The factor loading values were also acceptable. This is because the personal input subscale contains only one, which does not meet the requirement for conducting CFA, as at least two items are needed in a subscale.

According to the results, it indicates that the Indonesian version of the STEM-CIS can be used to measure: (1) all four disciplines as a single measure; (2) discipline-specific measure (e.g., only mathematics subscale); and (3) specific SCCT aspect (e.g., only self-efficacy subscale). Some recommendations are highlighted. First, it is not recommended to measure the SCCT aspect in only a specific discipline, since the personal input subscale was not fit in the model. However, it is still possible to use the questionnaire to measure the aspect of SCCT in only a specific discipline if the study did not include the personal input subscale as their measured scale. Second, the high RMSEA in the models of SCCT aspects might be because the items are task-specific. Clustering items from different disciplines into one SCCT subscale without considering the characteristic of respondents could lead to issue in the results. For instance, students might assess themselves as having high self-efficacy in mathematics but not in engineering. Furthermore, according to the results of exploratory factor analysis, some items (but not all) in the original questionnaire were not categorized under a single SCCT aspect in this study. Therefore, it is suggested to understand the characteristic of respondents before using the questionnaire with only a single specific SCCT subscale.

The reliabilities of the adapted STEM-CIS were high both as a single measure and as discipline-specific measures (0.898 ≤ α ≤ 0.95). This indicates that the adapted STEM-CIS provides a consistent and dependable measurement of STEM career interest both as a whole and within each discipline, ensuring that responses from Indonesian high school students accurately reflect their career aspirations in STEM. It is in line with previous studies by Ünlü et al. (0.86 ≤ α ≤ 0.93)^6^ and Mau et al. (0.92 ≤ α ≤ 0.94)^5^. However, the reliability values in this study were higher compared to the study results of Kier et al. (0.77 ≤ α ≤ 0.89)^12^. The reliability value of the science subscale is lower compared to other subscales that agree with previous findings^6,12^. Although the reliability values of the measure based on the SCCT subscales were lower than in the previous study^5^, they were still acceptable.

The adapted STEM-CIS has been shown to be psychometrically sound for each of the subscales of science, technology, engineering, and mathematics, the four-factor full subscales, and in each SCCT subscale. Hence, the questionnaire can be used in a variety of ways, to measure one single discipline, four subscales that work together as a single measure, and to measure one single SCCT subscale.

It should be considered that the different results from previous studies are possibly due to: (1) different age of the target respondent, e.g., Kier et al.^12^ targeted middle school respondent; (2) the characteristic of cultural and personal background of the respondents (e.g., nationality, major, ethnicity, SES); and (3) environmental factors (e.g., teaching and learning, parents, curriculum).

## Conclusion

The adapted STEM-CIS has shown good psychometric evidence as a single measure, as a discipline-specific measure, and as an SCCT-specific subscale measure, replicating the sound psychometric properties of the original English version of STEM-CIS. However, in the discipline-specific measures and the SCCT-specific subscale measure, the degrees of freedom were low that affect the neglect in the results of RMSEA. Despite this, the adapted STEM-CIS can be applied to Indonesian high school students.

This research highlights the critical role of thorough instrument adaptation in ensuring cultural and contextual relevance. By adapting STEM-CIS to the Indonesian context, potential issues such as linguistic misinterpretation, cultural bias, and reduced validity were reduced. This adaptation ensures that the instrument is meaningful and interpretable for the Indonesian high school population, enabling researchers to generate accurate and reliable data.

The study showed that STEM-CIS can be used as a foundation for other researchers to apply STEM-CIS to Indonesian high school students. The adapted STEM-CIS in Indonesian language can be used as a single questionnaire, as a separate discipline-specific questionnaire, and SCCT-specific subscale questionnaire. Furthermore, the results of validity and reliability can be used as a comparison for other researchers to validate the Indonesian version of STEM-CIS in a different sample background or for other researchers to validate STEM-CIS in a cross-cultural background, with careful consideration of language and contextual factors.

For practical and methodological implications, it suggests that the instrument can be used to guide future studies and interventions aimed at fostering STEM interest, particularly in underrepresented and diverse populations. The approach outlined here underscores the importance of cultural adaptation for advancing the validity and applicability of STEM-related research in diverse contexts.

This study also contributes to the STEM literature by validating a non-Western, context-specific tool for assessing STEM career interests, addressing the global need for culturally relevant measures. It provides empirical support for SCCT in a new cultural setting, reinforcing the role of self-efficacy, outcome expectations, and environmental influences in shaping career interests. Additionally, this study differentiates between discipline-specific and general STEM career interest, offering insight into how STEM career pathways may develop across various domains.

This study limited the sample diversity and location of the sample used. There was an unbalanced sample proportion according to gender, grades, and student majors. Furthermore, the sample did not come from all provinces of Indonesia, since it was concentrated only in the Java and Sumatera provinces. Moreover, this study did not investigate its differential item functioning (DIF) test to examine the behavior of the respondents to understand the items according to their gender, race, and school location. The potential bias that exists due to the nature of the self-reported assessment is also highlighted. There is the possibility that the instrument measures the expectations of parents of students rather than their interests. On the basis of these limitations, it is recommended that future studies employ the DIF test. Furthermore, the Indonesian version of STEM-CIS needs to be validated in a more diverse sample and proportional sample characteristics.

## Data Availability

The datasets generated and/or analysed during the current study are available in the Mendeley repository, 10.17632/pnnhy57kg9.1.
